# Transcriptomics in Toxicogenomics, Part I: Experimental Design, Technologies, Publicly Available Data, and Regulatory Aspects

**DOI:** 10.3390/nano10040750

**Published:** 2020-04-15

**Authors:** Pia Anneli Sofia Kinaret, Angela Serra, Antonio Federico, Pekka Kohonen, Penny Nymark, Irene Liampa, My Kieu Ha, Jang-Sik Choi, Karolina Jagiello, Natasha Sanabria, Georgia Melagraki, Luca Cattelani, Michele Fratello, Haralambos Sarimveis, Antreas Afantitis, Tae-Hyun Yoon, Mary Gulumian, Roland Grafström, Tomasz Puzyn, Dario Greco

**Affiliations:** 1 Faculty of Medicine and Health Technology, Tampere University, 33200 Tampere, Finland; pia.kinaret@helsinki.fi (P.A.S.K.); angela.serra@tuni.fi (A.S.); antonio.federico@tuni.fi (A.F.); luca.cattelani@tuni.fi (L.C.); michele.fratello@tuni.fi (M.F.); 2 BioMediTech Institute, Tampere University, 33200 Tampere, Finland; 3 Institute of Biotechnology, University of Helsinki, 00790 Helsinki, Finland; 4 Institute of Environmental Medicine, Karolinska Institutet, 17177 Stockholm, Sweden; pkpekka@gmail.com (P.K.); penny.nymark@ki.se (P.N.); grafstromrc@gmail.com (R.G.); 5 Division of Toxicology, Misvik Biology, 20520 Turku, Finland; 6 School of Chemical Engineering, National Technical University of Athens, 15780 Athens, Greece; irini.liampa@gmail.com (I.L.); hsarimv@central.ntua.gr (H.S.); 7 Center for Next Generation Cytometry, Hanyang University, Seoul 04763, Korea; hakieumy12@gmail.com (M.K.H.); gksakdma0529@gmail.com (J.-S.C.); taeyoon@hanyang.ac.kr (T.-H.Y.); 8 Department of Chemistry, College of Natural Sciences, Hanyang University, Seoul 04763, Korea; 9 Institute of Next Generation Material Design, Hanyang University, Seoul 04763, Korea; 10 QSAR Lab Ltd., Aleja Grunwaldzka 190/102, 80-266 Gdansk, Poland; k.jagiello@qsarlab.com (K.J.); t.puzyn@qsarlab.com (T.P.); 11 Faculty of Chemistry, University of Gdansk, Wita Stwosza 63, 80-308 Gdansk, Poland; 12 National Institute for Occupational Health, Johannesburg 2000, South Africa; natashaS@nioh.ac.za (N.S.); maryG@nioh.ac.za (M.G.); 13 Nanoinformatics Department, NovaMechanics Ltd., Nicosia 1065, Cyprus; melagraki@novamechanics.com (G.M.); afantitis@novamechanics.com (A.A.); 14 Haematology and Molecular Medicine Department, School of Pathology, University of the Witwatersrand, Johannesburg 2000, South Africa

**Keywords:** transcriptomics, toxicogenomics (TGx), high throughput, microarrays, sequencing, experimental design, engineered nanomaterials (ENM), toxicology, alternative risk assessment

## Abstract

The starting point of successful hazard assessment is the generation of unbiased and trustworthy data. Conventional toxicity testing deals with extensive observations of phenotypic endpoints in vivo and complementing in vitro models. The increasing development of novel materials and chemical compounds dictates the need for a better understanding of the molecular changes occurring in exposed biological systems. Transcriptomics enables the exploration of organisms’ responses to environmental, chemical, and physical agents by observing the molecular alterations in more detail. Toxicogenomics integrates classical toxicology with omics assays, thus allowing the characterization of the mechanism of action (MOA) of chemical compounds, novel small molecules, and engineered nanomaterials (ENMs). Lack of standardization in data generation and analysis currently hampers the full exploitation of toxicogenomics-based evidence in risk assessment. To fill this gap, TGx methods need to take into account appropriate experimental design and possible pitfalls in the transcriptomic analyses as well as data generation and sharing that adhere to the FAIR (Findable, Accessible, Interoperable, and Reusable) principles. In this review, we summarize the recent advancements in the design and analysis of DNA microarray, RNA sequencing (RNA-Seq), and single-cell RNA-Seq (scRNA-Seq) data. We provide guidelines on exposure time, dose and complex endpoint selection, sample quality considerations and sample randomization. Furthermore, we summarize publicly available data resources and highlight applications of TGx data to understand and predict chemical toxicity potential. Additionally, we discuss the efforts to implement TGx into regulatory decision making to promote alternative methods for risk assessment and to support the 3R (reduction, refinement, and replacement) concept. This review is the first part of a three-article series on Transcriptomics in Toxicogenomics. These initial considerations on Experimental Design, Technologies, Publicly Available Data, Regulatory Aspects, are the starting point for further rigorous and reliable data preprocessing and modeling, described in the second and third part of the review series.

## 1. Introduction

Toxicology aims to understand how various agents can cause harm to humans and the environment. Traditionally, this means extensive testing strategies including exposure to animal models and consequently observing the outcome in the means of physical symptoms, or other measurable parameters. In Europe, the legislative restrictions on animal testing [1] as well as the advent of ENMs led to the realization that conventional toxicity testing is no longer feasible. Toxicity assays have been developed originally for bulk chemicals and pharmaceuticals. Instead, ENMs and other advanced materials are a too broad and novel category to be tested in a similar manner as the more traditional substances. ENMs are defined as structures with at least one dimension smaller than 100 nanometers [2]. Thanks to their unique intrinsic properties, nanomaterials are increasingly utilized in several industrial fields. ENMs are known to interfere with the traditional assays, making conventional testing approaches less feasible [3,4,5]. Thus, new strategies are needed to keep up the pace of the new substances and materials entering the market as well as to support the reduction of animal experiments. To overcome this bottleneck in the testing of the adverse effects, TGx approaches have been suggested as a complementing method for traditional safety assessment [6,7]. This has led to the establishment of several prominent programs in the EU and worldwide aiming to generate omics-based big data. These could be used in systems biology-based approaches to build predictive models, simultaneously shifting the focus from in vivo animal testing towards in vitro methodologies and in silico modeling [8]. Studying gene expression changes in response to a foreign substance or a compound allows the building of a detailed picture of the organisms molecular levels of alteration and consequently understanding the MOA of the possible toxicant [7]. Many relatively mature methods for examining transcriptional changes already exist. Microarrays and RNA-Seq allow relatively thorough analyses of the transcriptome, and their costs have been steadily decreasing to a level where these assays are now accessible to most researchers. Moreover, large-scale public databases are constantly expanding when more and more data are being produced. The emergence of omics technologies has resulted also in the expansion of methods and tools to handle the complex and large data sets. What the community of toxicologists, biologists, computational scientists, and other professionals is still lacking are more unified and consistent strategies for experimental set-up and design, exposure conditions, and data analysis, all needed steps to efficient interpretation of the outcome and possible hazard identification. Only using harmonized methodologies and pipelines can aid achieving more comprehensive cooperation with regulatory agencies. In this review, we focus on how to create, handle and analyze global, large-scale transcriptomics data from a toxicity point of view, especially emphasizing the new era of nanomaterial research and hazard assessment.

Careful planning and study design are key elements for successful transcriptomics experiments. To obtain valid and unbiased data, as well as to prevent the inclusion of measurement uncertainties and data that misrepresents the changes in expression, several key steps need to be carefully planned and handled. To evaluate and ensure significant and reliable outcomes, robust statistical analyses with a proper amount of sample replicates and appropriate exposure time points and doses are essential together with proper reference (control) groups [9]. Most of all, recognizing the possible pitfalls in experimental design and statistical uncertainties is crucial. Furthermore, transparency and proper data recording are essential for subsequent data analysis and interlaboratory repeatability.

The existing literature about TGx methodologies is currently scattered and focused on particular facets of the workflow. In this review, we discuss all the relevant aspects of using transcriptomics in toxicogenomics, which could be utilized to build more robust strategies to further promote transcriptomics approaches also in regulatory assessment. To this end, in this review series we cover all the steps needed for performing successful toxicogenomics experimentation, from study design to robust and comprehensive computational approaches.

## 2. Replicates and Reference Samples

To afford reliable statistical power to the downstream analysis, the number of sample replicates is an important factor to consider before any further steps are taken. Some tools and methods have been suggested to estimate the statistical power of a transcriptomic experiment with respect to the number of samples included [10,11,12]. For example, in case of a microarray experiment aiming at identifying differentially expressed genes, the minimum sample size can be estimated by means of permutation method proposed by Tibishirami et al. [13]. This allows identification of the minimum number of samples required to achieve a specific sensitivity (the proportion of truly differentially expressed genes) at a specified false discovery rate level [14]. In the same way, for RNASeq analysis the compromise between the number of biological replicates and sequencing depth should be identified [15]. The price per sample varies depending on the technology [7]. Together with the possible lack of biological replicates especially of in vivo samples, the price is usually considered as a limiting factor in transcriptomics. In general, the larger the variance between the biological replicates, the more replicates are needed. For example, exposure to a genetically similar model organism, such as cell lines or inbred mouse strains, provides a relatively homogeneous genetic background with marginal bias derived from the individuals, allowing measurement of fewer replicates as compared to models with complex and varying genotype or growth conditions [16]. Patient samples, instead, possess a highly variable genetic background, and thus require correspondingly more replicates to confirm the measured changes. As with all experiments, in order to understand the change in gene expression due to the treatment or exposure, a baseline needs to be determined by comparisons against a negative reference group. It should be also kept in mind that, for example, gender or age might cause varying responses, especially when patient samples or animals are used to study transcriptional changes. New materials, such as ENMs, for example, bring additional challenges to the testing conditions. The number of replicates depends on the experimental setup and there are no universal standardized guidelines. Transcriptomics datasets derived from ENM-exposed samples deposited in the GEO database (https://www.ncbi.nlm.nih.gov/geo/) generally comprise a number of replicates ranging between 3 and 8. For example, Bajat et al. studied the gold nanoparticles (AuNP) toxicity on in vitro human models. In this study, Caco-2 cells were exposed to spherical gold nanoparticles of two different sizes at two different concentrations and two time points. They used three replicates for each experimental condition, and their results indicate that, at high concentrations, smaller AuNPs induce metal exposure and oxidative stress signaling pathways [17]. Moreover, Poulsen et al. [18] studied the inflammatory responses of two different multiwalled carbon nanotubes MWCNTs by having an experimental setup with three doses and three time points and five replicates. The same experimental set-up was used by Bourdon et al. [19] to investigate the effect of the exposure to carbon black (CB) nanoparticles in mice. Another example is the study performed by Dymacek et al. [20] on the effect of MWCNT in lung inflammation and pathogenesis. The analyzed dataset included four different doses and four time points with eight replicates for each experimental setup. As classical in vitro assays might be susceptible to interference originating from the unique physicochemical properties of ENMs (see section “Other technical considerations”), the most common control includes the addition of ENMs to the assay components alone, followed by measurement of the intrinsic fluorescence or absorbance of the ENMs [21]. Thereafter, additional controls are added, such as the inclusion of the ENM with the analyte, which is again measured, and so on. Additionally, comparison against positive reference might be beneficial for validating the experimental procedures [22,23]. Positive reference group could be for example a known toxic substance or bacterial component, depending on the expected outcome or cellular activation status. For example, in case of ENM, studying the toxic potential of hollow carbon nanotube structures, a positive or negative control could be an exposure to well-known substances [22,23,24]. Experimental planning is an integrated part of TGx analysis. In this context, given the interdisciplinary nature of TGx, it is crucial to consult experts from complementary fields to coherently link all the necessary steps of the experiment.

## 3. Time and Dose Selection

One major step in the toxicological assessment involves the outlining of the relationship between the amount of exposure and the biological response. Together with the selection of the appropriate model organism and a sufficient number of replicates, the doses and time points need to be carefully considered [25].

Dose selection for the TGx experiments largely depends on the exposure route (oral, airways, dermal, etc.), exposure frequency and duration, exposure system, as well as the model organism. Different computational models for ENM dose estimation in the respiratory tract of animals and for in vitro models have been developed [26,27]. As with other substances and pharmaceuticals, also in the case of ENM the possible toxic effects depend on several factors, and thus no universal guidelines exist for dose metrics. Poulsen et al. exposed mice with MWCNT by intratracheal instillation with 6, 18, and 54 μg per mouse [28], whereas Wallin et al. exposed mice with titanium dioxide nanoparticles with dose of 18, 54, and 162 μg per mouse [29]. Kinaret et al. instead, mimicked chronic exposure model by exposing mice by oropharyngeal aspiration for four consecutive days to 10 and 40 μg of MWCNT and by inhalation exposure for 4 hours a day for four consecutive days with 6.2–8.2 mg/m${}^{3}$ of MWCNT [30]. Moreover, Scala et al. exposed THP-1, A549 and BEAS-2B cells to eight different ENMs with 0.1, 0.5, 1, 5, 10, 50, 100, and 500 μg/mL for 48 hours and concluded that the concentration of 10 μg/mL induced low levels of cytotoxicity and high cell viability, enabling recording of the fine molecular changes caused by the ENM instead of the more dominant apoptotic and cellular stress signals [31]. In conclusion, the dose selection requires careful considerations and planning on the ENM-specific aspects and should be designed distinctively based on the research question.

In toxicology, the dose–response assessment is crucial in determining the dose thresholds in which a low-toxic substance becomes harmful, or a highly toxic substance does not cause harm. In more traditional dose–response assessment, to determine the highest dose, which does not cause statistically significant adverse effects, the No observed adverse effect level dose (NOAEL) can be used to determine the point of departure (POD) for the chemical. More recently, the benchmark dose (BMD) approach has been introduced, in which a dose–response model is fitted to the data and the response rate is recorded. These approaches are dependent on the dose selection and sample size. Traditionally, these methods require time- and resource-consuming methodologies of biological parameters such as tumor incidence, cell proliferation, or body weight changes. TGx, instead, provides faster strategies for determining BMD levels, with less screening and animal experiments [32]. Moreover, the goal of BMD is to build estimates about safe daily exposure levels, and for this, TGx approach can provide faster predictions allowing complex modeling of gene expression changes in thousands of features simultaneously. Further details on BMD modeling can be found in the third part of this review: “Transcriptomics in toxicogenomics, Part III: Data Modeling”.

Several efforts have been made to demonstrate that BMD values from transcriptional data are comparable with apical data [33,34,35,36,37]. TGx analyses are still relatively expensive; thus, a reasonable strategy to optimize between doses and specific end points of interest needs to be considered. Useful TGx profiles can be obtained by testing one dose in one time-point, although, in order to cover the boundaries of toxic effects (i.e., POD), more doses are required [38]. Nonetheless, one dose and one time point exposures will provide information about specific and direct MOA [39]. Alternatively, using multiple doses will help to disentangle complex mechanisms of dose-dependent molecular alteration but will not allow any interpretation of time course, or long-term effects. One dose with long term follow-up instead, will help to highlight the pathways of recovery. Multiple exposure time points, as well as doses, are beneficial in defining dose-dependent effects and transcriptional PODs [40]. To disentangle the kinetic patterns of molecular alterations exerted by an exposure, analyzing multiple time points becomes of paramount importance. As toxicity testing involves evaluation of acute phase response as well as possible chronic effects, exposure time points need to be cautiously considered in order to recognize the course of molecular activities. In general, microarrays and sequencing experiments provide information about a specific response at a given time. For this reason, one exposure time point results in only revealing a small fraction of the whole biological outcome. Additionally, enough dose and time points need to be measured in order to predict long-term toxic effects through advanced computational approaches and machine learning algorithms [41,42], as also discussed in detail in the third part of this review. Nonetheless, most of the gene expression changes are measurable in hours or days after exposure, rather than months or years. Studying the changes in expression profiles after relatively short exposure to toxicants is useful for investigating the relationships between acute and chronic mechanisms of toxicity. Furthermore, when relationships between exposure duration and the produced gene expression profiles are compared, secondary effects can be identified [43].

Other important issues that need to be considered are the comparable doses applied during the in vitro and in vivo studies. Before the comparison of the results, one needs to ensure that the tested in vitro doses are suitably equivalent for the in vivo situation [44]. Especially in the case of ENMs, the proper dose metrics are not easily defined, with surface area-to-volume ratio having a significant effect on ENMs properties and reactivity [45].

### 3.1. Exposures and Sample Quality

Following the ancient dictum “dose makes the poison” , when deciding whether an agent presents a toxicity concern, exposure assessment is central. Conversely, toxicity testing needs to be done at biologically realistic concentrations; otherwise, a hazard that is detected may not be meaningful. For thorough TGx analysis that aims at answering the questions about dose-dependency and safe limits, it is therefore important to consider multiple exposure doses. Recommendations for dose and endpoint selection, target organism, route and method of administration, and time of exposure are indicated in guidelines provided by regulatory entities like the European Commission’s science and knowledge service and the The Organisation for Economic Co-operation and Development (OECD) [46]. Before proceeding to the TGx experimentation, other measurements should be considered to fine-tune proper dose and time selection. For example, controlling viability, confluency, and cytotoxicity in in vitro models is recommended in order to avoid biased molecular effects caused by induced apoptotic signaling cascades and excess cellular stress caused by the growth conditions [47]. This might not be simple, as discussed in the subsequent paragraph “Other technical considerations”.

Chemical exposures for toxicological studies need to be performed in a highly controlled manner and with careful administration of the toxicant. Controlled exposure conditions with minimal effects derived from different environmental factors are crucial. Changes for example in temperature, pH, and agglomeration status are all having an impact on the outcome. Again, as an example, much discussion and arguments about proper testing methods for ENMs have risen due to properties such as hydrophobicity, charge, and reactivity, causing for example agglomeration, protein corona formation, and other unexpected behaviors in biological systems [48].

Furthermore, after successful exposure, before performing extensive microarray or RNAseq experiments, the quality of the obtained RNA needs to be verified. One important step in sample processing, together with the proper storage conditions, is the purification of the mRNA samples. It needs to be ensured that no contamination for example from genomic DNA or RNAse enzymes will interfere with the analysis. For this, spectrophotometric analysis gives an estimation of RNA purity and concentration. In order to confirm the integrity of the RNA molecules, more advanced measurements, such as microcapillary electrophoretic RNA separation, are recommended [49].

In toxicology, the route (exposure pathway) and duration of exposure, together with appropriate doses and chemical composition of the possible toxicant, affect the chosen exposure method and model organism. The principles of toxicology exposure can be found in guidelines from governmental institutions and the European Chemicals Agency (ECHA) and are outside the scope of this review.

### 3.2. Minimizing the Variation

Undesirable variation caused by technical and other non-biological sources are called batch effects. Well-known batch effects in transcriptomics data (further discussed in the second part of this review) include technical, environmental, and individual factors, such as changes in time, personnel, instruments, sample storage conditions and period, reagent slots, and slides/chips/wells. Technical artifacts and batch effects cannot be entirely avoided in high-throughput experiments, but careful design and sample randomization can reduce these effects significantly [50]. Therefore, batches and other sources of variation should be uniformly distributed across the biological groups [51]. In practice, after quality check, the samples need to be randomized in a controlled manner. This means, that, in order to reduce additional biases, samples, slides, and dyes are distributed (organized) to cover all possible sample combinations [52]. For example, if all control samples are labeled with the same dye, and treated samples with another dye, the additional effect caused by the labeling cannot be removed from the data. For this reason, carefully controlled randomization is a necessity. Additionally, if enough replicates are obtainable, some of the technical biases can be reduced by pooling several samples together before the experiment. Nonetheless, pooling should not be performed on biological samples, such as cancer tissue samples of patients, in which the individual differences are of high importance [25,53].

An often neglected part of transcriptional data management is the accurate recording of the metadata. This includes recording of all the possible sources of variation and batch effects such as changes in operators, exposure and procedure dates, sample storage and processing conditions, sample labeling, dyes, array and slot/position information, changes in reagent slots/batches, quality measurements, and other possible deviations. In order to ensure replicability of the experiment and the data analysis, accurate information about the samples need to be recorded and reported. For this, uniform phenotypic data files are essential, and need to be created as shown in the example in Table 1. Besides, major public databases such as the Gene Expression Omnibus (GEO) and ArrayExpress have adopted the Minimum Information About a Microarray Experiment (MIAME) and Minimum Information about a high throughput SEQuencing Experiment (MINSEQE) standards [54,55,56]. Thus, they offer full support for the submission of the genomic experiment data that these standards define as the minimum information that enables the interpretation and reproducibility of the results of relevant experiments. Brazma et al., when first proposed the MIAME documentation, argued that the initial gap in the information related to the quality and reliability of publicly available microarray data would affect not only the understanding of the outcome of their analysis but also any future effort to use the stored data for automated data analysis or mining. Furthermore, FAIR (Findable, Accessible, Interoperable and Reusable) Data Principle guidelines for data management, are highly recommended, allowing to promote the maximum reuse of research data (see section “Publicly available datasets for toxicogenomics” paragraph) [57]. Supplementary standardized information relevant to the exposure details can further facilitate the reusability of TGx data [58].

### 3.3. Model Systems

An urgent issue in substituting animal toxicity testing with in vitro experimentations has been raised by Poulsen et al. [44]. The same concern extends to transcriptomics studies [44,59,60]. Efforts have been made to assess the relevance of the *in vitro-*based gene expression changes (DNA microarray as well as RNA-Seq data) to in vivo health effects for chemicals [44,59,60,61]. The replacement of in vivo transcriptomics studies with in vitro tests would allow investigation of specific biomarkers and pathways in a less expensive, more ethical and time-effective manner. In vitro approaches further allow several time points and doses to be tested with a more reasonable time scale when compared to extensive, time-consuming in vivo experimentation.

Unfortunately, the task is not simple nor straightforward and, at present, in vitro models cannot fully replicate in vivo outcomes. For example, the comparison of the in vivo inhalation of rats with the in vitro exposures using three cell lines (rat lung epithelial cells, rat primary alveolar macrophages and co-culture of lung epithelial and primary alveolar macrophages) performed for five different types of nanoparticles indicates only a little correlation in their toxicological behavior [44]. Similar observations were reported by Warheit et al. and Sayes et al., where the authors assessed the predictability of the in vitro lung response induced by nano-sized particles (nanoscale zinc oxide and fullerenes) for in vivo pulmonary hazard potential [62,63]. The authors postulated that these differences may be caused by the fact that in vivo responses evaluate the toxicity in whole organisms while the in vitro primarily focuses on the response of a single isolated cell type [44,62,63,64]. However, on the opposite side, there are studies with a good agreement between the in vitro and in vivo models in case of the genotoxic potential of ENMs [65]. This might indicate that proper selection of the cell type as well as doses and toxicity endpoints would be the crucial factors influencing this comparison [44], although genotoxicity is a special case involving the singular and universal molecular target, DNA. According to recommendations by Poulsen et al., another important issue should be the choice of data analysis [44]. They observed that, besides the fact that the regulations of most individual genes measured in the in vivo and in vitro approaches are different, at the pathway level the common cellular functions (i.e., the oxidative stress and fibrosis) were activated. Similarly, Kinaret et al. demonstrated that when proper data analysis is utilized and the different ENM properties are taken into account, similarities in transcriptomics responses between mouse lung in vivo and human macrophage cell lines after ENM exposures can be observed [66]. Despite a little overlap between perturbed genes in vivo and in vitro was observed, similar molecular and cellular functions were identified. Thus, as a strategy, they proposed a network-based response modules that project the knowledge from individual genes to transcriptome-based biological functions [67]. In effect, they postulated that the proper strategy of data analysis gives the possibility to use the transcriptomic signature measured in vitro to characterize the outcome for the in vivo scenario [66]. Although some studies highlight the correlation between exposure systems, more effort is needed to unify the different aspects between the systems, such as doses and time points, as well as data handling and transcriptomics analyses. Advanced co-culture systems and 3D models are already extensively explored, and will provide more realistic comparisons also for toxicogenomics. In this manner it should be possible to gain a more complete appreciation of differences between in vitro and in vivo TGx outcomes.

### 3.4. Other Technical Considerations

Especially in the case of ENMs, many additional technical considerations should be taken into account. A way to control the quality of the data and to ensure correct measuring procedures is to validate the assay in order to assess whether or not the target of interest is actually being detected, i.e., instead of false-positive results. This is an important step related to standardization in data generation and analysis, since most optical signal-based assays are subject to a form of assay interference, which is inadvertently caused by the presence of ENMs within the cell or cytosol [21,68,69,70]. For example, the most common molecule used in molecular cell biology assays would be an enzyme, which is a protein. Ong and colleagues reported that ENMs can bind to proteins and dyes and alter their structure and/or function [71,72,73,74,75,76,77]. They went further to explain that this may adversely influence reactions and cause significant changes in enzyme activity, fluorescence, and/or the absorbance characteristics of indicator molecules [71,72,73,74,75,76,78,79]. Thus, the false positive and/or negative results in conventional screening assays are due to interference caused by intracellular ENMs that affect the color, chemical activity, light scattering, etc. [80,81,82].

Some studies have tried to compensate for this phenomenon by including more controls, or by verifying one method with another similar method. Examples include the testing of the cytotoxicity of ENMs such as SiO2 in A549 human alveolar epithelial cells, which was assessed by using two different toxicity assays [83,84,85,86]. As will be discussed below, microarray gene expression results are commonly validated by quantitative real-time PCR (qPCR). For example, the global transcriptomic analyses of exposures to surface-modified gold nanoparticles (Au-NPs) were investigated by Grzincic and colleagues. The authors tested gene expression in human dermal fibroblast (HDF) and prostate cancer (PIC3) cell lines via RNA microarray, which was confirmed via qPCR [87]. However, assay interference has been reported for various types of PCR [88,89,90]. Specifically, Li and colleagues reported a generalized hindrance of the PCR process [88]. Humes and colleagues concluded that assay interference occurred when the surface-oxidized multiwalled carbon nanotubes (oxMWCNTs) inhibited the reverse transcription step of RT-qPCR used to assess gene expression [88,89,90]. However, pristine MWCNTs did not cause the same level of interference, which emphasizes the role of surface chemistry in these types of analyses. Au-NPs were also reported to affect the reverse transcription efficiency in RT-qPCR [91]. Gao and colleagues later found that the efficiency of the high-fidelity DNA polymerase (Phusion) was significantly inhibited by some of the major types of metal oxide NPs (e.g., Fe2O3, ZnO, CeO2, FeO4, Al2O3, CuO, TiO2), but that this did not introduce mutations, i.e., the overall error rate was not significantly different and single nucleotide polymorphisms were not introduced [90]. The time-dependent quenching effect of ZnO NPs on fluorescence emissions was also investigated by Zhang and Lai [92]. These authors went further to determine the interference caused by other metal oxide nanoparticles in combination with the porphyrin quenching of ZnO NPs, i.e., an example of interactions between NPs in mixtures. These studies mentioned above support the need to assess the interactions between, especially metal oxide, intracellular NPs and genetic material, so as to explain the inconsistencies found in the literature regarding ENM-related TGx and/or toxicity.

In general, Omics techniques have been supposed to exhibit lower interference due to the removal of the ENMs during the isolation of the analyte [81]. Unfortunately, even if the ENMs are removed by repeated wash-steps within the isolation procedure, the damage might have already been done when intracellular or cell membrane-bound ENMs interfere with the biological molecules that are used to assess toxicity [93]. ENMs could also remain within the cells, or bound to the cell membranes [69,94]. Again, the recommendation is to test for the unintended interactions and to also validate results with multiple techniques that use different detection methods. In this manner, more stringent conditions for the use of these types of assays can be employed to prevent misinformation from being produced in nanotoxicity testing, which has substantial implications for our understanding of, as well as confidence in, the reported bioactivity of ENMs [21].

## 4. Transcriptomic Technologies in Toxicogenomics

### 4.1. Polymerase Chain Reaction, PCR

PCR is considered as a state-of-the-art method to study gene transcription of known sequences. The relative change in expression is measured by first synthesizing cDNA from the mRNA transcripts with reverse transcriptase enzyme and then using PCR to amplify the genes of interest. Quantitative PCR is a valuable assay for gene expression analysis, but has limits in number of analytes, enabling assay of only a limited number of genes at a time, making it time consuming and impractical from a toxicogenomics point of view. However, it is still important, and highly recommended to confirm and validate the obtained results from high content experiments. PCR-based multiplex methods have been developed, enabling the amplification of several sequences simultaneously. Nonetheless, from a toxicogenomics point of view, a genome-wide analysis is needed, for in-depth coverage of the changes in the whole genome.

### 4.2. DNA Microarray

DNA microarrays were first proposed by Schena et al. in 1995 [95]. In microarray technology, thousands of probes are fixed to a surface and samples (targets) are labeled with fluorescent dyes for detection after hybridization. Laser light is used to excite the fluorescent dyes and collect relative signal intensities. The hybridization intensity is represented by the amount of fluorescent emission, which gives an estimate of the relative amount of the different transcripts that are represented. mRNA from biological samples is reverse transcribed and labeled with one or more fluorescent dyes. After hybridization, fluorescence is measured separately and captured in two images. These are merged to produce a composite image, which goes through preprocessing before expression values are analyzed.

Microarrays have been widely applied in TGx studies and related predictive efforts [30,31,66,96,97,98,99]. DNA microarrays can capture relevant transcriptomic responses with concordant biological signals, independently from the platforms and laboratories [100,101]. A further advantage of microarray technology is that it is relatively mature, with numerous well-established commercial and open source data analysis tools [102,103]. Nonetheless, one of the limitations of this technology is the inability to detect unknown transcripts, as the probes are designed on the basis of known nucleotide sequences. Moreover, cross-hybridization events may occur when working with highly repetitive genomes, such as human’s, resulting in an inaccurate observed expression value of the gene compared to the real expression [104]. From the TGx point of view, distinct sample handling and data preprocessing steps might limit the reproducibility of the results in different laboratory environments.

### 4.3. RNA-Sequencing

Although the advent of high-throughput hybridization-based technologies, such as DNA microarrays, significantly boosted the generation of large scale gene expression profiles, the recent advances in sequencing technologies further improved such capability. In fact, the so-called Next-Generation Sequencing (NGS) technologies are currently employed in multiple fields of the life sciences, including the toxicogenomics. In this context, the RNA-sequencing (RNA-Seq) allowed overcoming the technical limitations imposed by the hybridization-based techniques. For instance, the RNA-Seq allows the detection of gene expression with increased dynamic range, solving the problem of probes saturation for highly expressed transcripts [105]. Furthermore, it does not need a priori knowledge of the genomic sequence of the studied organism and it does not suffer from the above mentioned cross-hybridization events, especially in the analysis of complex genomes. As a consequence, RNA-Seq allows to perform a de novo transcript discovery in order to 1-identify unannotated transcripts, and 2-characterize new transcripts generated by alternative splicing [106]. Conversely, an appropriate analysis plan should be employed in order to avoid or mitigate certain biases that could occur during the data management and analysis. For instance, previous works showed that standard normalization procedures can affect the sensitivity of the differential expression analysis, reflecting the behavior of a relatively small number of either high-count or ubiquitous genes [107,108]. RNA sequencing typically produces bigger and more complex data, which need longer time and more sophisticated analytical approaches, as compared for instance with DNA microarray experiments. Although TGx experiments are increasingly employing transcriptome profiling, the analysis pipelines are still far from being standardized. To date, a benchmark of the optimal analytic procedures in the transcriptome profiling in TGx experiments has not been formulated. More recently, the reduction of the costs for the analysis of a single transcriptome made possible the accomplishment of big scale studies, carried out by international programs, such as CMAP, TOX21, and LINCS1000 aimed at understanding the responses of hundreds of cell models upon treatment with several classes of chemicals/compounds.

### 4.4. Single Cell RNA-Sequencing

Since the first single-cell RNA sequencing (scRNA-Seq) protocol was published in 2009 [109], there has been increasing interest in applying this technique in various areas of research, especially in cellular heterogeneity, such as in tumors [110], the immune system[111], the brain [112], and embryo development [113], which is often masked in the typical RNA-Seq. In TGx studies, this technique has been extensively used to analyze drug tolerance in cancer cells[114]. The scRNA-Seq, as its name indicates, analyses the transcriptome of an individual cell and is mainly used to resolve the transcriptional heterogeneity and to identify rare cell populations that would otherwise be hidden in bulk-cell analyses [115,116,117]. Currently, there are approximately 20 different scRNA-Seq protocols that have been reported to address various research purposes.

Full-length methods, such as SMART-seq, cover the whole transcriptome and thereby increase the number of mappable reads as well as the detected genes per cell. This is useful for analyzing genes that are expressed at low levels [118]. However, one major drawback of full-length methods is that they do not support multiplexing and thus require intensive cost and labor as each sample should have their own sequencing library. In contrast, 3’-end counting methods, such as Drop-seq, MARS-seq, and CEL-seq, only cover the 3’-end of the transcripts [119]. The transcripts, in this case, can be indexed with barcodes and unique molecular identifiers (UMIs) to allow multiplexing of samples and quantification of gene expression. However, as the reads are limited to the 3’-end of the transcripts, the overall number of detected genes per cell is lower than in full-length methods. In terms of single-cell isolation, all these technologies separate single cells into nanoliter reaction chambers. In the droplet-based methods, each cell is partitioned in a hydrogel droplet [120]. In microfluidics-based methods, cells are individually captured on a microfluidic chip, whereas in plate-based methods, individual cells are placed into wells on a 96-well plate by micropipetting or fluorescence-activated cell sorting [121]. Of all the scRNA-Seq sample preparation platforms, 10X Genomics’ Chromium is one of the most widely used commercial solutions. It is a droplet-based 3’-end counting system. On the 10X Genomics’ Chromium, each cell is captured in a water-in-oil emulsion droplet that contains a gel bead labeled with sequencing adapters, 16-nucleotide barcodes for cell identification, 10-nucleotide UMIs for transcript identification, and poly[T]primers for mRNA capture.

Although there are various scRNA-Seq methods available for different research questions, most of them adhere to a similar workflow. The first and most important step is the isolation of viable, single cells from the tissue of interest. Next, the isolated individual cells are lysed and the mRNA molecules are captured using the poly[T]-primed oligomers that bind to the poly[A] tail of the mRNA. Analysis of non-poly[A]-tailed mRNA will require more specialised protocols [122,123]. The complementary DNA (cDNA) is then synthesized by reverse transcription. Depending on each method, the reverse transcription primers can have additional nucleotide sequences (i.e., barcodes, UMIs, etc.). Subsequently, the cDNA is amplified to acquire an adequate amount for sequencing by either PCR, or in vitro transcription. Then, libraries are prepared by pooling and fragmenting the amplified cDNA. Libraries are finally sequenced via NGS. The preprocessing, analysis, and interpretation of the data is a challenging research topic on its own and will be further discussed in later parts.

### 4.5. High-Throughput Transcriptomics

High-throughput transcriptomics technologies dramatically increase the throughput of gene expression data generation. In particular, they allow a low-cost profiling of dose responses. These methods are very diverse and include microarray-based, RNA sequencing-based as well as other technologies. For example, the L1000 (Library of Integrated Network-Based Cellular Signatures 1000) platform that is used in the LINCS CMAP database uses flow cytometry and labeled beads [124]. It measures only approximately 1000 genes, but these carefully selected “landmark genes” can be used to predict the expression of nearly 11,000 genes. This illustrates the principle of redundancy and co-regulation within gene expression. The S1500+ platform designed by the US National Toxicology Program has 2753 probe sets or genes, including all the L1000 genes [125]. The larger number of genes is used to improve the accuracy of gene estimation and to measure directly many toxicologically relevant and pathway-associated genes. The S1500+ platform uses a TempO-Seq targeted RNA-Seq method [126]. Compared to the 10-20 million counts in a regular RNA-Seq, it only needs 0.5-3 million reads per profile. An example of an array-based high-throughput platform is the Affymetrix/ThermoFisher Scientific Clariom GO Screen assay that includes approximately 20,000 gene ontology-related genes in a 384-multiplex format. In general, the same or very similar methods can be used to analyze high-throughput and regular expression experiments. If the data is continuous or array-based, microarray methods and RNA-Seq methods with count data are recommended. Due to the larger volume of data, automated R/Bioconductor or other workflows need to be run [127]. Here, tools such as eUTOPIA (A solUTion for Omics data PreprocessIng and Analysis) can be helpful in reducing the coding workload [128].

## 5. Publicly Available Datasets for Toxicogenomics

The scientific advancement in the toxicogenomics field is strongly dependent on the availability of high-quality TGx datasets. Thus, in the last years, many efforts have been made to produce good quality databases such as Chemical Effects in Biological Systems (CEBS) [129,130], Connectivity Map (CMAP) [131], LINCS 1000 [124], DrugMatrix [132], Open TG-GATEs[98], ArrayExpress [133], and Gene Expression Omnibus (GEO) [54,134].

CEBS is a comprehensive toxicogenomics resource compiled by the National Center for Toxicogenomics (NCT) within the National Institute of Environmental Health Science (NIEHS). It includes the study design, the timeline, the clinical chemistry, and histopathology findings, as well as microarray and proteomics data from in vivo and in vitro exposure [130]. CEBS freely available online at http://tools.niehs.nih.gov/cebs3/ui/.

The CMAP project started in 2006 with gene expression profiles of 164 small-molecule compounds and was later updated to "build 2", containing gene expression profiles from three cultured human cells treated with 1309 bioactive small molecules, aimed at finding connections among small molecules sharing a mechanism of action, chemical and physiological processes, diseases, and drugs [131].

The L1000 Connectivity Map is a Library of Integrated Network-based Cellular Signatures (LINCS) and was created as a large-scale expansion of the original CMAP, in which almost 20,000 small-molecules have been profiled on up to 77 cell lines at 96 hours after the knockdown [124]. The experiments were performed by using the L1000 platform, which aims to capture the greatest amount of variation while measuring only a subset of 978 genes, named “landmark genes” [124]. This subset of genes was chosen since they are able to capture the greatest proportion of the variance in expression [124,135,136].

The DrugMatrix dataset contains gene expression response to compound treatments in rat tissues [132]. Approximately 600 different compounds have been profiled in up to seven different tissues of rats, representing over 3200 different drug–dose–time–tissue combinations. The dataset also provides histopathological, hematologic and clinical chemistry data associated with compound treatments, allowing specific forms of toxicity to be investigated. The DrugMatrix dataset is available through the CEBS database using the CEBS Guided Search tools [130].

Open TG-GATEs is a TGx database that stores gene expression profiles and traditional toxicological data derived from in vivo (rat) and in vitro (primary rat hepatocytes, primary human hepatocytes) exposure to 170 compounds at multiple doses and time points [98]. The gene expression data for a test compound were derived from the administration of individual compounds at up to four dose levels and eight time-points (corresponding to four single-dose studies and four repeated-dose studies).

ArrayExpress repository stores data from high-throughput functional genomics experiments, and provides these data for reuse to the research community [133].

Gene Expression Omnibus (GEO) is a public repository for high-throughput microarray and next-generation sequencing data sets, carried out by the research community. The resource supports archiving of raw data, processed data and metadata which are indexed, cross-linked, and searchable. All data is freely available for download in a variety of formats [54,134].

As introduced above, usable, high-quality databases for TGx data are already available. In order to deal with the growing number of large datasets and to be able to fully utilize the potential of data sharing in toxicology, more comprehensive and integrated infrastructure should be still created to handle the data according to the FAIR principles. In practice, this requires proper metadata handling, unique identifiers, trusted repositories for easy access and data handling, clear usage licenses, and opportunities for interoperability [57].

## 6. Regulatory Aspects

The concept of systems biology has been known already for decades, but the emergence of high-throughput methodologies and omics approaches rapidly expanded in the beginning of the 21st century. Since then, transcriptional profiling has become a well recognized field of bioscience. Due to reducing costs and well-established infrastructure, today omics approaches are available for a wide research community. The ample amount of available omics-based exposure data enables researchers to combine and interpret complex and large datasets in a meaningful way. Additionally, in Europe, directives on animal testing limitations and marketing restrictions for example in case of cosmetics, encourage the development of alternative testing methods.

Toxicogenomics is recognized as a promising approach to characterize biological activity caused by exposure to different stressors. For example, in the ECHA’s Guidance "Information Requirements and Chemical Safety Assessment", TGx-related technologies and toxicity testing are discussed in the context of in vitro data and Adverse Outcome Pathways (AOPs). The guidance states that the value of the in vitro models in toxicity testing could be increased by incorporating molecular based markers through the application of proteomic and toxicogenomic approaches [137]. Also the European Food Safety Authority (EFSA) has recognized the potential of TGx [138]. Moreover, the REACH (Registration, Evaluation, Authorisation and Restriction of Chemicals) regulation emphasizes the utilization of TGx approaches in hazard assessment:

*“The Commission, Member States, industry and other stakeholders should continue to contribute to the promotion of alternative test methods on an international and national level including computer supported methodologies, in vitro methodologies, as appropriate, those based on toxicogenomics, and other relevant methodologies. The Community’s strategy to promote alternative test methods is a priority and the Commission should ensure that within its future Research Framework Programmes and initiatives such as the Community Action Plan on the Protection and Welfare of Animals 2006 to 2010 this remains a priority topic. Participation of stakeholders and initiatives involving all interested parties should be sought [139].*”

Although ongoing effort is being made in Europe to bring TGx approaches as part of toxicity and hazard assessment, no formal guidance yet exists. Nonetheless, there are several ongoing projects to produce frameworks for omics data-based risk assessment: In the European Union, the European Centre for the Validation of Alternative Methods (ECVAM), an integral part of the Joint Research Centre (JRC) and Extended Advisory Group for Molecular Screening and Toxicogenomics (EAGMST) in OECD are working towards TGx approaches for example by using the concept of AOP [140,141]. OECD supports the development and improvement of AOPs with TGx approaches, by filling the data gaps and supporting the available mechanistic toxicity data. In 2012, the OECD established a new programme on the development of Adverse Outcome Pathways. Since the beginning of the programme in 2012, OECD has released 16 publications on AOP, from which seven were published in the year 2019 [142]. In addition, the European Centre for Ecotoxicology and Toxicology of Chemicals (ECETOC) has together with ECVAM developed a Transcriptomics Reporting Framework for omics data processing and analysis [140]. The framework does not dictate the methodology to be used, but rather lays the basis for what should be reported when performing bioinformatics with the aim of supporting risk assessment. Thus, the current three-article series provides a basis for refining and extending the framework with guidelines based on the suggested state-of-the-art methods and approaches. Importantly, we suggest here a shift in thinking regarding the current focus on differentially expressed genes in the framework, as will be discussed in detail in Part II of this article series. Analysis of differentially expressed genes is often coupled to arbitrary *p*-value limits and fold-change thresholds, which leads to one of the largest sources of bias within TGx, i.e., the use of high irrelevant doses.

In the U.S., The National Toxicology Program (NTP), an interagency program composed by the National Institute of Environmental Health Sciences (NIEHS) of the National Institutes of Health (NIH), the National Center for Toxicological Research (NCTR) of the Food and Drug Administration (FDA), and The National Institute for Occupational Safety and Health (NIOSH) of the Centers for Disease Control and Prevention (CDC), has established a toxicogenomics testing program consisting of 5-day rodent chemical exposure studies (https://ntp.niehs.nih.gov/whatwestudy/testpgm/toxicogenomics/index.html—Retrieved 3 March 2020). All the TGx data generated by this collaboration are stored in the CEBS database and publicly available to the community. The agencies across continents are also collaborating in scientific advisory boards towards legitimate validation and international harmonization of testing strategies based on alternative test methods including TGx [143].

Data for adverse effects and toxicity are classically retrieved from epidemiological studies of humans or more commonly from experimental systems including in vitro cell culture models and in vivo animal models. The data generated by traditional toxicity tests are the cornerstone for human health risk assessment, determined from the identified changes and phenotypic effects. Nonetheless, new chemicals, and especially the new era of ENMs, require extensive testing and screening, for which conventional toxicity testing is not optimal. This problem has been widely recognized, and efforts for integrated approaches together with high-throughput methods and 3R principles aim to identify early molecular markers and pathways of toxicity. Instead of traditional endpoints, TGx provides a possibility to test the entire genome of a cell or tissue, and thus, reveal earlier effects leading to toxic outcomes [40].

Toxicogenomic approaches are supported in EU-programmes such as Framework Programme 7 (FP7) and Horizon 2020 (H2020), but to the best of our knowledge, no specific legislation exists for TGx approaches. Currently, toxicogenomics approaches can be used to complement more conventional methods. Required testing methods depend on the chemical and its composition, and in general, requirements for pharmaceuticals are more demanding than for industrial chemicals and require extended testing related to efficacy, safety, and quality control. In the European Commission’s Technical Guidance Document on Risk Assessment, the use of methods such as quantitative structure–activity relationship (QSAR) in human health risk assessment is recognized as a potentially contributing factor only for certain endpoints [144]. Moreover, the guidance states that modeled data should only be used for risk assessment purposes where it is supported by other strands of evidence [145].

Understandably more investigations are needed to further embed TGx-based evidence in regulatory decision making. International unification and harmonization of methods and data generation and sharing is extremely important in developing toxicogenomics approaches for hazard assessment [146]. Furthermore, in addition to regulatory implementation of TGx, targeting the industry with the methods as support for safety assessment at early stages of technological innovation and development (aiming for so called safe by design strategies) may support uptake into the generally more slow-adapting (as compared to industrial processes) regulatory processes (https://doi.org/10.1002/smll.201904749).

## 7. Conclusions

In this first part of the review, we consider the crucial aspects needed to perform unbiased and reliable toxicogenomics analysis. We discuss the necessary steps for designing the experiments, deciding the sample groups, timepoints and doses. Furthermore, we discuss the public databases and transcriptomics related technologies. TGx has a great potential to overcome the limitations of traditional hazard assessment and to reduce the amount of animal experimentations. Additionally, when repeatable and reliable transcriptomics results are achieved, TGx approach allows in vitro methods to be utilized in regulatory toxicology. This includes better utilization of known methods such as AOPs and dose-response assessment, as well as better characterization of the biological processes related to QSAR models.

Reliable TGx data are the starting point to elucidate compounds MOA and predicting toxicity. Even though public TGx datasets can be found, toxicogenomics advancement would benefit from the availability of more comprehensive gene expression data and toxic endpoint annotations. Open data access, open protocols and publicly available meta-data annotations are also important to make data findable, accessible, interoperable and reusable (FAIR Data Principles) [57].

In conclusion, TGx methods are already favored by a wide community of researchers and pharmaceutical industries to characterize the molecular mechanisms underlying toxicity by the means of AOPs and to further predict compound toxicity. Furthermore, the field is evolving and currently working to reduce animal tests by replacing them with in vitro experiments. Developing TGx towards regulatory decision making depends on multiple technical and conceptual solutions that are currently coming together [6,147]. It is difficult to say when these methods will replace animal tests, but they can already be used as convincing supporting evidence. Altogether, TGx will have a strong impact on in vitro-based workflows in risk-assessment, on drug development strategies and on clinical decision making.

## Figures and Tables

**Table 1:** Example of the phenodata table reporting.

Sample	Sample ID	Date	Material	Dose	Time	Day	Array.N	Hybr Date	Slot	Slid Bar Code	Operator
12	121	12/03/2019	$TiO_{2}$	10	24	Cy5	2	18.3.	1_2	757480412533_S05	PKI

## Abbreviations

The following abbreviations are used in this manuscript:

AOP	adverse outcome pathways
BMD	benchmark dose
CDC	Centers for Disease Control and Prevention
CEBS	Chemical Effects in Biological Systems
cDNA	complementary DNA
CMAP	Connectivity Map
EAGMST	Extended Advisory Group for Molecular Screening and Toxicogenomics
ECHA	European chemicals agency
ECVAM	European Centre for the Validation of Alternative Methods
EFSA	European Food Safety Authority
eUTOPIA	A solUTion for Omics data PreprocessIng and Analysis
FAIR	Findable, Accessible, Interoperable and Reusable Data Principle
FDA	Food and Drug Administration
GEO	Gene Expression Omnibus
GO	gene ontology
GTEx	Genotype-Tissue Expression
JRC	Joint Research Centre
L1000	Library of Integrated Network-Based Cellular Signatures 1000
MIAME	Minimum Information About a Microarray Experiment
MINSEQE	Minimal Information about a high throughput SEQuencing Experiment
MOA	mechanism of action
NCT	National Center for Toxicogenomics
NCTR	National Center for Toxicological Research
NIEHS	National Institute of Environmental Health Sciences
NIH	National Institutes of Health
NIOSH	The National Institute for Occupational Safety and Health
NTP	The National Toxicology Program
OECD	Organisation for Economic Co-operation and Development
TG-GATE	Toxicogenomics Project-Genomics Assisted Toxicity Evaluation System
PCR	polymerase chain reaction
POD	point of departure
QSAR	quantitative structure activity relationship
REACH	Registration, Evaluation, Authorisation and Restriction of Chemicals
RNA-Seq	RNA sequencing
scRNA-Seq	single-cell RNA sequencing
TGx	toxicogenomics
3R	Replacement, Reduction and Refinement in animal testing
